# Immunohistochemical Expression of p16 and CDK4 in Soft Tissue Tumors

**DOI:** 10.7759/cureus.35713

**Published:** 2023-03-03

**Authors:** Mala Sagar, Rita Yadav, Pankaj Deval, Madhu Kumar, Malti K Maurya, Sumaira Qayoom

**Affiliations:** 1 Department of Pathology, King George's Medical University, Lucknow, IND; 2 Department of Pathology, Prasad Institute of Medical Sciences, Lucknow, IND

**Keywords:** adipocytic tumors, immunohistochemistry, cdk4, p16, soft tissue tumors

## Abstract

Background and Objectives: The aim of this study was to determine the immunohistochemical expression of p16 (p16^INK4a^) and cyclin-dependent kinase 4 (CDK4) and CDK4 markers in various lineages of soft tissue tumors and to evaluate their role in differentiating atypical lipomatous tumors/well-differentiated liposarcomas from benign lipomas.

Material and Methods: A total of 70 cases of both excisional and incisional biopsies of soft tissue tumors were included in this study. Histopathological examination was done by using formalin-fixed, paraffin-embedded tissue samples. After that, we performed IHC expressions of p16 and CDK4 markers on the unstained slides of these soft tissue tumors.

Results: Immunohistochemical study showed that positive expressions of p16, CDK4, and combined (p16+CDK4) markers were 51.4%, 10.0%, and 12.9%, respectively in soft tissue tumors. Positive p16 expression was observed among a higher proportion of malignant cases (66.7%) as compared to benign (20.0%) and intermediate (50.0%) cases. This difference was found to be statistically significant (p=0.009). Negative expression of only CDK4 and combined (p16 and CDK4) were observed among a higher proportion of benign as compared to malignant and intermediate cases (90.0% vs. 78.6% & 75.0%, p=0.393 and 65.0% vs. 26.2% & 37.5%, p=0.028, respectively). This difference was not found to be statistically significant. For adipocytic tumors, the majority of malignant and intermediate tumors had positive p16 (7/7; 100%) and CDK4 (6/7; 85.7%) immunohistochemical expression. These differences were found to be statistically significant.

Conclusion: Immunohistochemical marker p16 can be used to differentiate between malignant and benign soft tissue tumors. Amongst adipocytic tumors, combination of p16 and CDK4 immunohistochemical expression can be used to differentiate liposarcomas from benign ones

## Introduction

Soft tissue tumors are a diverse group of tumors that are classified as benign or malignant on the basis of the line of differentiation. Benign tumors closely resemble normal tissue and have a limited capacity for autonomous growth. They show little tendency of local invasion and have a low rate of local recurrence after conservative therapy. Malignant tumors, also known as sarcomas, are aggressive tumors and are capable of invasion, recurrence, and distant metastasis [1].

Soft tissue sarcomas have been classified according to a histogenetic concept. For example, fibrosarcoma arising from fibroblasts, and osteosarcoma arising from osteoblasts. However, it seems clear that most sarcomas arise from primitive multipotential mesenchymal cells, which in the course of neoplastic transformation undergo differentiation along one or more lines [2]. Sarcomas are rare malignant tumors of mesenchymal origin, that arise in connective tissue, in contrast to the more frequent and better-known carcinomas of epithelial origin [3]. Soft tissue sarcomas, compared with carcinomas and other neoplasms, are relatively rare and constitute fewer than 1.5% of all cancers with an annual inci­dence of about six per 100,000 persons [4].

The pathogenesis of most soft tissue tumors is still unknown. The main causes are various physical and chemical factors and exposure to ionizing radiation. These may be inherited or acquired immunologic defects. Trauma is also a cause of sarco­mas. Rarely, soft tissue sarcomas can arise in scar tissue, at fracture sites, and in the vicinity of plastic or metal implants, usually after a latent period of several years [5].

p16 is a tumor suppressor gene that is an important cell cycle regulator. It inhibits cell cycle progression by binding to cyclin-dependent kinases (CDKs) 4/6, preventing the inactivation of the retinoblastoma protein. Cyclins and their catalytic partners, CDKs, regulate the cell cycle in eukaryotic cells. The progress of the G1 phase of the cell cycle is stimulated by G1 cyclins, such as cyclin D and cyclin E. For instance, cyclin D forms a complex with CDK4, and this complex plays an important role in propelling the cell cycle through the G1 checkpoint, a critical phase of cell proliferation [6]. CDK4 and p16 are useful ancillary diagnostic tools to differentiate well-differentiated and dedifferentiated liposarcoma from other adipocytic tumors [7].

In this study, we analyze the immunohistochemical expression of CDK4 and p16 in the various lineages of soft tissue sarcomas and evaluate their role in differentiating atypical lipomatous tumors/well-differentiated liposarcomas from benign lipomas.

## Materials and methods

This was a prospective cross-sectional study conducted from September 2018 to August 2019, in the Department of Pathology, King George’s Medical University (KGMU), Lucknow, India, after getting approval from the university's Institutional Ethics Committee (approval number: 646/ethics/19). A total of 70 cases of soft tissue tumors including benign, intermediate, and malignant, were received in the Department of Pathology, KGMU, in the one-year study period. Inclusion criteria include histologically diagnosed cases of soft tissue tumors, patients who gave consent to enroll in the study, and availability of clinical details at presentation. Exclusion criteria include inadequate biopsy tissue for immunohistochemistry, patients who were not willing to give consent to be a part of the study, cases in which tissue may be lost during antigen retrieval, and poorly preserved tumor tissue.

We took cases of soft tissue sarcoma and benign lipoma that were diagnosed histologically and immunohistochemically in the Department of Pathology, KGMU. Both excisional and incisional biopsies were studied. Tissue specimens were collected and immediately fixed in 10% formalin. 3-5 micron thick paraffin-embedded sections were stained with hematoxylin and eosin (H&E) stain for histopathological examination. All sarcomas were reported as per the Fédération Nationale des Centres de Lutte Contre le Cancer (FNCLCC) grading system, by a group of pathologists. After that, 3-4-micrometer-thick sections were taken from each block for both p16 and CDK4 immunohistochemical (IHC) staining on 3-Aminopropyltriethoxysilane-coated slides. Staining and evaluation were done by using mouse anti-human p16 monoclonal antibody (Clone MX007) and mouse monoclonal anti-human CDK4 antibody (Clone DCS-35) as per standard protocol. Tonsil served as a positive control for p16. Diagnosed case of liposarcoma served as a positive control for CDK4. For negative control, primary antibody was omitted while performing immunostaining. Both positive and negative controls were included in every batch of IHC staining.

**Figure 1:**
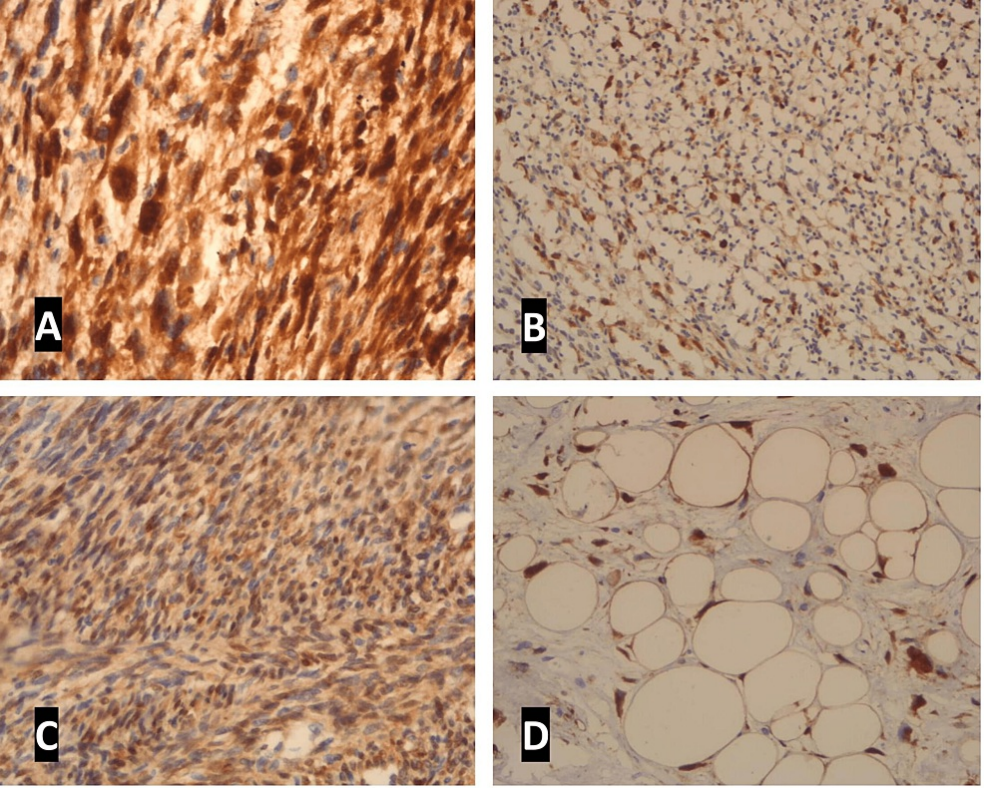
(A) Immunohistochemistry P16 was diffusely and strongly expressed in malignant and intermediate soft tissue tumors like leiomyosarcoma (x400); (B) Synovial sarcoma (x200); (C) Dermatofibrosarcoma protuberance (x400); (D) CDK4 was diffusely and strongly expressed in atypical lipomatous tumor (x200 ).

Immunostainings were evaluated by two independent pathologists. Immunoreactivity scores for both p16 and CDK4 expression were given based on the percentage of tumor cells showing only well-defined nuclear staining and cytoplasmic staining was disregarded. (Table 1). IHC expression of p16 and CDK4 in various tumors is shown in Figure 1.

**Table 1:** Immunoreactivity scores for both p16 and CDK4 immunohistochemistry marker IHC: immunohistochemistry

Points	IHC expression	Percentage of tumor cells
0	Negative	≤5% nuclear staining of tumor cells
1	Focal positive	6% - 20% nuclear staining of tumor cells
2	Diffuse positive	21% - 100% nuclear staining of tumor cells

The statistical analysis was done using IBM SPSS Statistics for Windows, Version 21.0 (Released 2012; IBM Corp., Armonk, New York, United States). Data measurements were presented as mean and standard deviation. Chi-square test was used for the intergroup comparison of enumeration data. Analysis of variance (ANOVA) test was used to compare the within-group and between-group variances among the study groups. P <0.05 was considered to indicate a statically significant difference

## Results

Out of 70 cases of soft tissue tumors enrolled in the study, 42 (60.0%) were histopathologically found to be malignant, 20 (28.6%) were benign, and the remaining (11.4% ) were intermediate, according to the WHO classification of soft tissue tumors. In our study, 15 cases of adipocytic tumors were also taken.

The most common diagnosis among malignant cases was synovial sarcoma (21.4%) followed by leiomyosarcoma (19.0%), undifferentiated pleomorphic sarcoma (11.9%), and fibromyxoid sarcoma (11.9%). Less common diagnoses were Ewing’s sarcoma, dedifferentiated liposarcoma, and rhabdomyosarcoma (9.5% each), and one (2.4%) case each of chondrosarcoma, malignant peripheral nerve sheath tumor (MPNST), and myxoid liposarcoma (Table 2).

**Table 2:** Distribution of malignant cases according to diagnosis (n=42)

SN	Diagnosis	No. of cases	Percentage
1.	Chondrosarcoma	1	2.4
2.	Ewing's sarcoma	4	9.5
3.	Fibromyxoid sarcoma	5	11.9
4.	Leiomyosarcoma	8	19.0
5.	Dedifferentiated liposarcoma	4	9.5
6.	Malignant peripheral nerve sheath tumor	1	2.4
7.	Myxoid liposarcoma	1	2.4
8.	Rhabdomyosarcoma	4	9.5
9.	Synovial sarcoma	9	21.4
10.	Undifferentiated pleomorphic sarcoma	5	11.9

Out of 20 benign tumors, lipoma was the most common diagnosis (25.0%) followed by angiomyolipoma, benign fibromyxoid neoplasm, calcifying fibrous tumor, and fibroma (15.0% each), and one (5.0%) case each was diagnosed as dermatofibroma, hemangioma, and myxoma (Table 3).

**Table 3:** Distribution of benign cases according to diagnosis (n=20)

SN	Diagnosis	No. of cases	Percentage
1.	Angiolipoma/angiomyolipoma	3	15.0
2.	Benign fibromyxoid neoplasm/fibrous histiocytoma	3	15.0
3.	Calcifying fibrous tumor	3	15.0
4.	Dermatofibroma	1	5.0
5.	Fibroma	3	15.0
6.	Hemangioma	1	5.0
7.	Lipoma	5	25.0
8.	Myxoma	1	5.0

Among histopathologically intermediate cases, the most common diagnosis was dermatofibrosarcoma protuberans (DFSP) (62.5%) followed by atypical lipomatous tumor (25.0%) and low-grade myofibroblastic sarcoma (12.5%) (Table 4).

**Table 4:** Distribution of Intermediate cases according to diagnosis (n=8)

SN	Diagnosis	No. of cases	Percentage
1.	Dermatofibrosarcoma protuberans	5	62.5
2.	Atypical lipomatous tumor	2	25.0
3.	Low-grade myofibroblastic sarcoma	1	12.5

The IHC expression of p16, CDK4, and combined (p16+CDK4) markers in various lineages of soft tissue tumors are shown in Table 5. Positive p16 expression was observed in 36 cases (51.4%) while negative p16 expression was observed in 31 cases (44.3%) and the remaining three cases were found to be focal positive (4.3%). Out of 36 p16 positive cases, malignant, benign, and intermediate were 28, four, and four, respectively. Negative CDK4 expression was observed in the majority of the cases (81.4%) while positive CDK4 expression was observed in 10.0% of cases; the rest were found to be focal positive (8.6%). Both p16 and CDK4 positive expression was observed in only 12.9% of cases, 38.3% had a negative expression for both, and the rest 48.6% cases had positive expression either for p16 or CDK4.

**Table 5:** IHC expression of p16, CDK4, and combined (p16+CDK4) markers IHC: immunohistochemistry *combined p16+CDK4 marker

IHC expression	p16		CDK4		p16 + CDK4*	
	No.	Percentage	No.	Percentage	No.	Percentage
Negative/both negative*	31	44.3	57	81.4	27	38.6
Positive/both positive*	36	51.4	7	10	9	12.9
Focal positive	3	4.3	6	8.6	–	–
Either positive*	–	–	–	–	34	48.6
Total	70	100	70	100	70	100

The association of p16 IHC expression and histopathological findings of soft tissue tumors is shown in Table 6. Negative p16 expression was observed among a higher proportion of benign tumors (75.0%) as compared to malignant (31.0%) and intermediate (37.5%) while positive p16 expression was observed among a higher proportion of malignant tumors (66.7%) as compared to benign (20.0%) and intermediate (50.0%) while focal positive expression was observed in a higher proportion of Intermediate tumors (12.5%) as compared to benign 5.0%) and malignant cases (2.4%). This difference was found to be significant statistically (c²=13.409; p=0.009).

**Table 6:** Association of p16 (IHC) expression and histopathological finding IHC: immunohistochemistry

Histopathological finding	Total	Negative p16		Positive p16		Focal Positive p16	
		No.	Percentage	No.	Percentage	No.	Percentage
Benign	20	15	75.0	4	20.0	1	5.0
Malignant	42	13	31.0	28	66.7	1	2.4
Intermediate	8	3	37.5	4	50.0	1	12.5

The association of CDK4 IHC expression and histopathological findings of soft tissue tumors is shown in Table 7. Negative CDK4 expression was observed among a higher proportion of benign cases (90.0%) as compared to malignant (78.6%) and intermediate (75.0%) cases while positive CDK4 expression was observed among a higher proportion of intermediate (25.0%) as compared to benign (5.0%) and malignant (9.5%) cases while focal positive expression was observed in a higher proportion of malignant cases (11.9%) as compared to benign (5.0%) and intermediate cases (0.0%). This difference was not found to be significant statistically significant (c²=4.100; p=0.393).

**Table 7:** Association of CDK4 (IHC) expression and histopathological finding IHC: immunohistochemistry

Histopathological finding	Total	Negative CDK4		Positive CDK4		Focal Positive CDK4	
		No.	Percentage	No.	Percentage	No.	Percentage
Benign	20	18	90.0	1	5.0	1	5.0
Malignant	42	33	78.6	4	9.5	5	11.9
Intermediate	8	6	75.0	2	25.0	0	0.0

The association of combined p16+CDK4 IHC expression and histopathological findings of soft tissue tumors is shown in Table 8. Negative expression in both p16 and CDK4 was observed in a higher proportion of benign tumors (65.0%) as compared to malignant (26.2%) and intermediate cases (37.5%) while a higher proportion of malignant (57.1%) as compared to benign (35.0%) and intermediate cases (37.5%) had positive expression in either p16 or CDK4; positive expression in both p16 and CDK4 was observed in a higher proportion of intermediate (25.0%) cases as compared to benign (0.0%) and malignant (16.7%). This difference was not found to be significant statistically significant (c²=10.852; p=0.028).

**Table 8:** Association of combined p16+CDK4 (IHC) expression and histopathological finding IHC: immunohistochemistry

Histopathological finding	Total	Negative (p16+CDK4 )		Positive (p16+CDK4 )		Either p16 or CDK4 positive	
		No.	%	No.	%	No.	%
Benign	20	13	65.0	0	0.0	7	35.0
Malignant	42	11	26.2	7	16.7	24	57.1
Intermediate	8	3	37.5	2	25.0	3	37.5

IHC expression of p16, CDK4, and combined (p16+CDK4) markers in adipocytic tumors as shown in Table 9. For adipocytic tumors, the majority of the benign tumors had a negative expression of p16 (6/8; 75%) and CDK4 (8/8; 100%) while the majority of malignant and intermediate tumors had positive p16 (7/7; 100%) and CDK4 (6/7; 85.7%) expression. These differences were found to be significant statistically (p16: c²=8.750; p=0.003; CDK4: c²=11.429; p=0.001).

**Table 9:** Expression of p16 and CDK4 in adipocytic tumor EP: either (p16 or CDK4) positive; FP: focal positive

Diagnosis	Total	p16			CDK4			Both P16 and CDK4		
		Percentage Negative	Percentage Positive	Percentage FP	Percentage Negative	Percentage Positive	Percentage FP	Percentage EP	Percentage Negative	Percentage Positive
Benign										
Angiolipoma/ angiomyolipoma	3	2 (66.7)	1 (33.3)	0 (0.0)	3 (100.0)	0 (0.0)	0 (0.0)	1 (33.3)	2 (66.7)	0 (0.0)
Lipoma	5	4 (80.0)	0 (0.0)	1 (20.0)	5 (100.0)	0 (0.0)	0 (0.0)	1 (20.0)	4 (80.0)	0 (0.0)
Malignant										
Dedifferentiated Liposarcoma	4	0 (0.0)	4 (100.0)	0 (0.0)	1 (25.0)	2 (50.0)	1 (25.0)	1 (25.0)	0 (0.0)	3 (75.0)
Myxoid liposarcoma	1	0 (0.0)	1 (100.0)	0 (0.0)	0 (0.0)	1 (100.0)	0 (0.0)	0 (0.0)	0 (0.0)	1 (100.0)
Intermeddiate										
Atypical lipomatous tumor	2	0 (0.0)	2 (100.0)	0 (0.0)	0 (0.0)	2 (100.0)	0 (0.0)	0 (0.0)	0 (0.0)	2 (100.0)

## Discussion

Soft tissue tumors, especially sarcomas, cannot be differentiated on clinical and histomorphological grounds alone; there are many techniques to categorize them correctly. These techniques are IHC, cytogenetics, and molecular genetics. IHC is used to identify the different lineages of mesenchymal tumors. IHC also plays a major role in soft tissue tumor classification, diagnosis, treatment, and prognosis.

p16 is a tumor suppressor gene and it is an important cell cycle regulator. It inhibits cell cycle at G1-S checkpoint by binding to cyclin-dependent kinases 4/6 and preventing the inactivation of the Rb protein. p16 may be mutated or deleted in many cancers [7]. p16 gene maps to chromosome 9p2l-22. It is a region that is frequently deleted or rearranged in diverse types of cancer cells [8,9], and has been investigated in mesenchymal neoplasms, but with varying conclusions. Some studies report that p16 alterations are infrequent in soft tissue sarcomas [10], whereas others report that altered expression of p16 protein was seen in 94% of sarcoma cases [11]. CDK4 is an oncogene whose product inhibits RB1 by phosphorylation and it inactivates E2F resulting in loss of the G1-S checkpoint. It is expressed in different types of normal and neoplastic cells and overexpressed in some epithelial and mesenchymal tumors like liposarcoma, osteosarcoma, malignant peripheral nerve sheath tumor in addition to rhabdomyosarcoma [12,13].

In the present study, we have examined the IHC expression of p16 and CDK4 in soft tissue tumors. In our study, 70 histologically diagnosed cases of soft tissue tumors were included of which 14 (20%) were incisional and 56 (80%) were excisional biopsies. Out of 20 benign cases, 15 (75%) showed negative p16 expression and five (25%) cases showed positive expression (four diffuse and one showing focal positivity). In these five cases, one in three cases of benign fibrous histiocytoma, one in three of fibroma, one in five cases of lipoma, one case of hemangioma, and one case of angiomyolipoma were positive for p16 nuclear expression. Previous studies also showed focal p16 expression in 60% of cases of angiomyolipoma [9]. In a study by Kammerer-Jacquet et al., 14 cases came out to be positive for p16 expression in 44 cases of angiomyolipoma [14].

Out of 42 malignant cases, 28 (66.7%) cases were positive for p16 expression. In eight intermediate cases, four (50%) cases were diffuse positive and one (12.5%) case was focally positive. This association came out to be statistically significant (p=0.009). Similar results were obtained in various other studies where they studied the expression of p16 in various lineages of sarcomas [15,16]. For IHC methods, we adopted the technique of Thway et al., where for each antibody, only well-defined nuclear reactivity was considered positive, and staining that was ill-defined, perinuclear, or cytoplasmic was disregarded [15]. We got five cases of liposarcomas in our study which included four dedifferentiated liposarcomas and one myxoid liposarcoma variant. All of these showed p16 expression. In many previous studies, it was found that p16 was overexpressed in well-differentiated, undifferentiated, and myxoid liposarcomas, and p16 was proposed as a diagnostic marker [14,15,17].

Ninety percent of benign cases were negative for CDK4 expression, and 10% of cases that showed positive expression were calcifying fibrous tumors (2/3 cases). In 42 malignant cases, nine (21.4%) cases showed CDK4 positivity. Out of these nine cases, the majority (4/9, 44.4%) were various liposarcomas (three dedifferentiated liposarcomas and one myxoid liposarcoma), with the remaining being two cases of undifferentiated pleomorphic sarcoma, one case of fibromyxoid sarcoma, one case of rhabdomyosarcoma, and one case of synovial sarcoma indicating that positive CDK4 expression is more prevalent in various liposarcomas as compared to other sarcomas. Weber-Hall et al. also observed that tumors originating from smooth muscle and striated muscle cells show a gain of 12q13-15 chromosomal region or amplification of CDK4 as reported in leiomyosarcoma and rhabdomyosarcoma [18]. In intermediate soft tissue tumors, two out of eight cases showed CDK4 positivity and both cases were of Atypical lipomatous tumors. Thway et al. observed similar results in their study [15]. Our results showed that CDK4 is a good marker to differentiate benign adipocytic tumors from well-differentiated and dedifferentiated liposarcomas, which was also observed in other studies [13,15].

In 20 benign cases, none showed positivity for both p16 and CDK4. Out of 42 cases of malignant tumors, seven cases (16.7%) showed positive expression for both markers. In these positive cases, the majority (4/7, 57.1%) were of various liposarcomas (three well-differentiated liposarcomas and one myxoid liposarcoma) along with one case of fibromyxoid sarcoma, one case of synovial sarcoma, and one case of undifferentiated pleomorphic sarcoma. Out of eight cases of intermediate sarcomas, two (25%) were positive for both p16 and CDK4, both of which were cases of atypical lipomatous tumors.

Liposarcomas have a supernumerary ring and giant marker chromosomes, which are composed of amplified material from the long arm of chromosome 12 (q13-15), in which several oncogenes are located and leading to overexpression of CDK4 [13,19].

In our study, 15 cases of adipocytic tumors were included. Of these, eight cases were benign and seven cases were intermediate and malignant. In benign cases, six (75%) were p16 negative whereas all eight cases were CDK4 negative. In the malignant and intermediate cases, seven were p16 positive while six out of seven were CDK4 positive. This indicates that a combination of p16 and CDK4 is helpful in differentiating benign lipomatous tumors from atypical and dedifferentiated lipomatous tumors. This association came out to be statistically significant (p16: p=0.003; CDK4: p=0.001). Previous studies showed that IHC expression of p16, CDK4, and MDM2 in adipocytic tumors found that a combination of CDK4 and p16 had even better discriminatory power to differentiate well and dedifferentiated liposarcomas from lipoma and also found that p16 was the most sensitive and specific among the three markers [15,20].

Limitations of the study

The study was conducted in a short time span of one year due to which the cases included did not represent the actual population. Hence, the number of included cases was also less. The correlation of p16 and CDK4 expression with overall survival in various soft tissue tumors could not be studied because of an inadequate follow-up period. Thus, further studies can be carried out to obtain much more confirmatory results.

## Conclusions

In this study, IHC expression of p16 was found to be more prevalent in malignant tumors as compared to benign soft tissue tumors. It can thus be used to differentiate between these two categories. CDK4 expression was seen more in malignant adipocytic tumors as compared to benign adipocytic tumors and other non-adipocytic soft tissue tumors. Thus, CDK4 can help differentiate between malignant and benign adipocytic tumors. In adipocytic tumors, the combination of p16 and CDK4 IHC expression can differentiate liposarcomas from benign tumors.
